# A nationwide cross-sectional study of 15,611 lesbian, gay and bisexual people in China: disclosure of sexual orientation and experiences of negative treatment in health care

**DOI:** 10.1186/s12939-020-1151-7

**Published:** 2020-04-01

**Authors:** Yiu-tung Suen, Randolph Chun Ho Chan

**Affiliations:** 1 Gender Studies Programme, The Chinese University of Hong Kong, Room 250, 2/F, Sino Building, Shatin, Hong Kong; 2 Department of Special Education and Counselling, The Education University of Hong Kong, Tai Po, Hong Kong

**Keywords:** Sexual and gender minorities, Sexuality, China, Social discrimination

## Abstract

**Background:**

Lesbian, gay and bisexual (LGB) people often face individual- and system-level barriers in health care. However, LGB people’s experiences of health care in non-European and non-American settings have been scarcely studied. In China, while it has been estimated that there are at least 70 million gender and sexual minorities, there has been no larger-scale study on LGB people’s experiences of health care beyond a focus on gay men and HIV. This study is the first larger-scale quantitative study to investigate LGB people’s experiences of health care in China, where non-heterosexuality is officially silenced and the needs of non-heterosexual people are largely ignored by service providers.

**Methods:**

An online survey was designed in joint partnership by academic, community groups and the United Nations Development Programme. Targeted and snowball sampling was adopted for participant recruitment. Such unique cross-sectoral partnership made this research possible in the authoritarian state of China where data collection on LGB people is extremely rare. For the analysis in this paper, a sample of 15,611 Chinese LGB people were included. Frequency and descriptive statistics were conducted to describe the LGB respondents’ demographic characteristics and their experiences in health care settings. Chi-square tests were conducted to test how experiences vary across LGB people with different demographic characteristics.

**Results:**

More than three quarters of the respondents said they would be willing to disclose to their medical care providers their sexual orientation if asked. However, only 5.7% of the respondents said that medical care providers ever asked them about their sexual orientation. About 8.0% of the LGB people surveyed reported having experienced negative treatment in medical care settings. Six percent (5.7%) of the Chinese LGB people said in accessing mental health care services, they were recommended, coaxed into, or provided conversion therapy for sexual orientation, gender identity or gender expression.

**Conclusions:**

There is a strong need to enhance LGB cultural competence among health care providers. Policymakers in China should also formulate laws, policies, regulations, clearly articulated codes of conduct, and transparent procedures and practices to ensure non-discrimination of LGB people in the health care system, with a particular focus on banning conversion therapy.

## Introduction

Lesbian, gay, bisexual, transgender and intersex (LGBTI) people often face individual- and system-level barriers to access health care services [1]. International health organizations increasingly recognize such barriers that LGBTI people face. The World Health Organization (WHO) has acknowledged that LGBTI people often face barriers to health care that profoundly affect their overall health and well-being [2]. WHO and other United Nations agencies have thus called on urgent responses by health care providers, among other stakeholders, to address the discriminatory laws and practices that LGBTI people face [3]. This paper focuses on the access to and experiences of health care among lesbian, gay and bisexual (LGB) people.

At the individual level, LGB people experience heterosexism – the prevailing social assumption that everyone is presumed heterosexual until proven otherwise, in society in general as well as health care settings. LGB people has reported negative treatment ranging from disrespectful language to harassment and discrimination [4, 5]. Upon revelation of patients’ LGB identity, health care professionals often display homophobic reactions, including embarrassment, anxiety, condescension, unfriendliness and hostility [6]. In the US, more than half of the LGB respondents have encountered unequal treatment within health care institutions [7].

At the system level, medical and nursing professionals often lack adequate awareness, knowledge and skills to work with LGB patients [8]. Many health care service providers are not trained to provide care for LGB people. In the US, as little as 16% of the surveyed academic medical institutions reported having comprehensive LGB-competence training for medical physicians [9]. Research consistently documented that medical service providers reported a lack of knowledge of and sensitivity to the unique health risks and needs of LGB people [10].

Given the presence of those barriers, LGB people are more vulnerable to unmet health needs [11], lower health service utilization [12], refusals of care [13], and delayed or substandard care [14] than non-LGB people. Specifically, LGB people expressed fear of being stigmatized and discriminated due to prior experiences of mistreatment in health care settings, which results in hesitation, avoidance and postponement of care [15]. Even in care, LGB people may conceal their sexual orientation and withhold their personal information from service providers in order to shield against hostile responses [16]. Health care providers who are unaware of their patients’ sexual orientation may also fall short of educating them about relevant health issues. Petroll and Mosack [17] found that primary care providers were less likely to recommend HIV testing and hepatitis A or B vaccination if they did not know their male patients had same-sex partners. With the dearth of timely and quality care, it may increase the chance of serious health issues going undetected and developing into chronic health conditions [18].

While the need to address such issues faced by LGB people in health has been increasingly recognized in some European and North American societies, by governments, health care providers, and the LGB civil society, the development to address LGB health in other parts of the world remains haphazard. Among many other reasons such as political, social, cultural, and religious factors, one of the reasons is a lack of empirical data to demonstrate the need to address LGB people’s experiences of health care. Many governments have refused to collect data on LGB people and instead claim that there are no specific health care needs of LGB people. It then also renders the LGB civil society difficult to argue for LGB-inclusive care. Thus, studies on LGB people’s experiences of health in non-European and non-American settings have largely been qualitative and smaller in scale. For example, a qualitative study by Ibragimov and Wong [19] found that gay and bisexual men in Tajikistan, Central Asia were refused health care services. The gay and bisexual men noted that service providers often made humiliating remarks related to their sexual orientation and asked questions about sexual life in an intimidating way [19]. Another qualitative study in Vietnam found that health care workers showed reluctance and discomfort when asking about and discussing sexual orientation and sexual behaviour with their gay and bisexual patients [20]. In a study of 161 women in Argentina, the majority of participants reported visiting a health care provider in the past year, but only a small number (17.2%) indicated that their health care providers had asked them their sexual orientation [21]. Other studies mainly discussed LGB people’s experiences of health care only in relation to sexual health and/or HIV issues [22, 23].

### Chinese context and LGB health

China is one such an example where understanding of LGB people’s experiences of health care has remained largely underdeveloped. Although it has been estimated that there are at least 70 million gender and sexual minorities in China [24], there has been no larger-scale study on LGB people’s experiences of health care.

LGB people’s experiences of health care needs to be contextualized in the cultural understanding of non-heterosexuality in Chinese society. It has been argued that homoerotic practice enjoyed great tolerance in ancient Chinese literature, and same-sex behaviours were socially permitted as long as the man maintained his social obligations to the family (i.e., getting married and bearing children). Since the Qing Dynasty (AD 1644–1911) and the impact of modernity, Chinese society has turned to Western bio-medical knowledge to understand sexual behaviours [25]. Homosexuality was classified as a sexual disorder in the first version of the Chinese Classification of Mental Disorders (CCMD) in 1978 [26]. Moreover, (mainly male) homosexuality had been increasingly associated with (and thus penalized as) a type of liumang zui (‘hooliganism’) when hooliganism was introduced in Article 160 of the Criminal Law in 1979 [27]. The removal of hooliganism law took place in 1997. Homosexuality and bisexuality were eliminated from the third edition of the CCMD in 2001. Nonetheless, the manual still retains a diagnosis of “ego-dystonic sexual orientation”, which suggests that “a person could be conflicted or suffering from mental illness because of their sexuality, and that condition could be treated” [28]. Since then, it has been argued that the Chinese government adopts a “not encouraging, not discouraging, and not promoting” attitude toward homosexuality in China [29]. The only ‘official’ recognition of the ‘existence’ of male homosexuals from the government was in 2003 due to the AIDS epidemic [30]. There has been government censorship of homosexuality content in films and television, and an attempt of imposing online censorship on Weibo, one of the largest social media platforms in China, in early 2018. This political climate makes LGB people in China highly vulnerable to discrimination and unequal treatment, in different social settings including health care.

There is no legal protection in China against discrimination on the ground of sexual orientation in service provision, including medical service. It has been reported that many mental health workers in China still consider homosexuality as sexual perversion and do not comply with the changes in CCMD, and subject their lesbian, gay and bisexual clients to conversion therapy, electric shock treatment, and aversion therapy in the guise of treatment for their ego-dystonic homosexuality and bisexuality [31]. Some LGB people were even involuntarily hospitalized or admitted to psychiatric wards due to their sexual orientation [32]. This is both partly based on the belief that homosexuality can be cured, but also partly motivated by some health workers’ wish to make money in a health care system that has transformed from state-funded to market competition driven in a matter of a few decades’ time [33].

There has been a dearth of systematic investigation of experiences of accessing physical and mental health care services among LGB people in China. A qualitative study by Feng et al. [34] revealed that stigma and discrimination from health care personnel were a main reason for underutilization of needed health care among gay and bisexual men. Most other health-related research on LGB people in China focused on gay men and centred on HIV/AIDS issues.

### Aims and research questions

This study is the first larger-scale quantitative study to investigate Chinese LGB people’s experiences of health care. It aims to explore the attitudes toward and experiences of LGB people in accessing health services in China.

Under the Chinese context, this research seeks to address four research questions:

1. Are LGB people in China willing to disclose their sexual orientation to their medical and mental health care providers?
2. To what extent do LGB people in China encounter negative treatment in medical and mental health care settings?
3. Do LGB people suppress their gender expression when receiving medical and mental health care services?
4. Which subgroups of LGB people are more likely to disclose their sexual orientation, encounter negative treatment and suppress their gender expression in medical and mental health care settings?

## Study data and methods

### Design

A nation-wide survey of LGBTI people was designed in joint partnership by academic, community groups and the United Nations Development Programme (UNDP). The survey covered all major provinces in China. It was the first larger-scale quantitative study to examine the health care utilization experiences among LGBTI people in China.

Targeted and snowball sampling was adopted for participant recruitment [35]. The online survey was distributed through 24 community organizations working with sexual and gender minorities across different parts of China, a number of educational institutions, Weibo and WeChat (two widely used online communication channels in China), LGBTI social networks, and UNDP’s social media accounts. Participants were invited to forward the survey to their contacts on social network platforms. People who expressed initial interest in the study were instructed to read the background and purposes of the study. They were asked to provide informed consent prior to the commencement of the study. Participants could complete and submit the survey in person on paper, online or through mobile phone.

The survey consisted of questions relating to the experiences of using medical and mental health care services in China. Specifically, participants were required to indicate whether they were willing to disclose their sexual orientation to their medical care providers and whether they were inquired about their sexual orientation by their medical care providers. They were also asked to indicate whether they encountered negative treatment and suppressed their gender expression when receiving medical care services. Since mental health care is less available and accessible than medical care in China, participants were asked whether they had received counselling/therapy (individually or in a group setting) for their mental health. For those who had received counselling/therapy, they were asked whether they were willing to disclose their sexual orientation to their mental health care providers (e.g., counsellors), encountered negative treatment and suppressed their gender expression when receiving mental health care services.

A total of 18,088 LGBTI people participated in the present study. For the analysis in this paper, participants who (1) were below the age of 18, (2) identified as transgender, non-binary or intersex, and/or (3) living in Hong Kong, Macau, Taiwan, and overseas were excluded, yielding a sample of 15,611 people.

Based on previous studies [36, 37], transgender people face unique stresses and challenges related to their gender identity and expression, which are clearly different from the experiences of LGB people. For instance, their gender identities are often not properly acknowledged and recognized in the health care settings, resulting in postponement and denial of care. In addition, transgender people have multiple and complex health care needs (e.g., gender confirmation surgery, access to pubertal blockers, and gender-affirming hormones) that are not shared by LGB people [38] The results of transgender and intersex people will be presented in a different publication in order to give a more in-depth discussion of their needs and challenges faced.

### Data analysis

Frequency and descriptive statistics were conducted to describe the demographic characteristics and show the prevalence of service utilization, sexual orientation disclosure, gender expression, and negative treatment among LGB people in medical and mental health care settings. Chi-square tests were performed to examine whether there were any significant differences in service utilization, sexual orientation disclosure, gender expression, and negative treatment between LGB people with different demographic characteristics (i.e. gender, sexual orientation, age group, living area, education level, and income level).

### Ethical considerations

Given the sensitivity of the topic, research protocols and measures were developed to ensure that data collection efforts cause no harm and fully respect the rights of LGB people, including their privacy and dignity. The data was anonymized to protect the confidentiality of the participants. The study was approved by the research ethics committee of the UNDP Being LGBTI in Asia in China and Peking University before data collection.

## Study results

### Participant demographics

A total of 15,611 LGB people in mainland China completed the study. Table 1 describes the demographic characteristics of the participants. Around three-fourth of the participants (74.2%) were male, while 25.8% were female. 76.3% of the participants were lesbian or gay, followed by bisexual (16.3%), pansexual (3.3%), asexual (2.2%), and questioning (1.9%). More than two-third (67.6%) were between 18 and 24, followed by 25–39 (30.3%) and 40 or above (2.1%). The sample was geographically diverse with respondents from all 31 provincial-level administrative divisions in China (not including Hong Kong, Macau, and Taiwan). Most participants were from Guangdong province (11.0%), Beijing municipality (10.0%), Jiangsu province (6.1%), and Sichuan province (6.0%). Majority of them lived in city (80.4%) and had college or above education (80.8%). Around half of the participants had an annual income of less than RenMinBi10,000 (USD1,450) (53.1%). While all the participants had the experiences of using medical care, only 11.1% of the participants had received mental health care.

**Table 1:** Demographic characteristics of the participants (*N* = 15,611)

	n (%)
Gender	
Male	11,583 (74.2%)
Female	4028 (25.8%)
Sexual orientation	
Lesbian / Gay	11,910 (76.3%)
Bisexual	2549 (16.3%)
Pansexual	516 (3.3%)
Asexual	338 (2.2%)
Questioning	298 (1.9%)
Age group	
18–24	10,551 (67.6%)
25–39	4727 (30.3%)
40 or above	333 (2.1%)
Living area	
City	12,544 (80.4%)
Town	2381 (15.3%)
Rural area	686 (4.4%)
Education level	
Middle school or below	649 (4.2%)
High school	2346 (15.0%)
College or above	12,616 (80.8%)
Income level	
Less than RMB10,000 (Less than USD1,450)	8282 (53.1%)
RMB10,000 – RMB49,999 (USD1,450 – USD7,249)	3925 (25.1%)
RMB50,000 – RMB99,999 (USD7,250 – USD 14,499)	2288 (14.7%)
RMB100,000 or above (USD14,500 or above)	1116 (7.1%)

### Medical care

#### Sexual orientation disclosure

Health care staff may need to ask the patient about their sex life or sexual partners in order to record their medical history or assist diagnosis. It was found that only 24.9% of the Chinese LGB people surveyed said they would not disclose to their doctor their sexual orientation if asked, 57.0% said it depends, whereas only 18.1% said they would. Results of chi-square tests indicated that those who were younger (*p* < .001), lived in the city (*p* < .001), were pansexual and asexual (*p* < .001), and had higher level of income (*p <* .001), were more willing to disclose their sexual orientation to their medical care providers (see Table 2).

**Table 2:** Experiences of using medical care services (*N* = 15,611)

	Sexual orientation disclosure			Being inquired about sexual orientation		Negative treatment		Suppress gender expression	
	Yes	It depends	No	Yes	No	Yes	No	Yes	No
Overall sample	18.1%	57.0%	24.9%	5.5%	94.5%	8.0%	92.0%	69.9%	30.1%
Gender									
Male	18.2%	56.1%	25.8%	7.0%	93.0%	8.4%	91.6%	73.9%	26.1%
Female	18.0%	59.5%	22.5%	1.0%	99.0%	6.9%	93.1%	58.3%	41.7%
	*p* < .001			*p* < .001		*p* = .002		*p* < .001	
Sexual orientation									
Lesbian / Gay	17.8%	56.3%	25.8%	6.4%	93.6%	8.7%	91.3%	72.6%	27.4%
Bisexual	17.1%	58.4%	24.4%	2.6%	97.4%	5.6%	94.4%	62.3%	37.7%
Pansexual	25.2%	60.1%	14.7%	2.1%	97.9%	5.0%	95.0%	58.3%	41.7%
Asexual	24.3%	57.7%	18.0%	2.1%	97.9%	7.4%	92.6%	56.2%	43.8%
Questioning	18.5%	62.8%	18.8%	2.3%	97.7%	8.1%	91.9%	60.7%	39.3%
	*p* < .001			*p* < .001		*p* < .001		*p* < .001	
Age group									
18–24	19.5%	60.6%	19.9%	4.8%	95.2%	7.5%	92.5%	67.7%	32.3%
25–39	15.4%	49.7%	35.0%	7.1%	92.9%	9.0%	91.0%	73.9%	26.1%
40 or above	14.1%	43.8%	42.0%	5.4%	94.6%	11.1%	88.9%	82.3%	17.7%
	*p* < .001			*p* < .001		*p* = .001		*p* < .001	
Living area									
City	18.7%	57.1%	24.2%	5.5%	94.5%	7.5%	92.5%	68.8%	31.2%
Town	15.6%	55.9%	28.5%	5.3%	94.7%	9.9%	90.1%	74.8%	25.2%
Rural area	16.3%	57.0%	26.7%	5.7%	94.3%	12.0%	88.0%	73.5%	26.5%
	*p* < .001			*p* = .92		*p* < .001		*p* < .001	
Education level									
Middle school or below	15.7%	55.8%	28.5%	7.7%	92.3%	12.6%	87.4%	74.4%	25.6%
High school	17.7%	56.3%	26.0%	5.9%	94.1%	9.9%	90.1%	73.6%	26.4%
College or above	18.3%	57.1%	24.5%	5.3%	94.7%	7.4%	92.6%	69.0%	31.0%
	*p* = .09			*p* = .02		*p* < .001		*p* < .001	
Income level									
Less than RMB10,000 (Less than USD1,450)	19.1%	60.4%	20.5%	4.6%	95.4%	7.4%	92.6%	67.9%	32.1%
RMB10,000 – RMB49,999 (USD1,450 – USD7,249)	16.8%	55.8%	27.4%	6.4%	93.6%	9.6%	90.4%	72.1%	27.9%
RMB50,000 – RMB99,999 (USD7,250 – USD 14,499)	15.8%	51.9%	32.3%	6.7%	93.3%	8.0%	92.0%	72.2%	27.8%
RMB100,000 or above (USD14,500 or above)	20.1%	46.1%	33.9%	6.6%	93.4%	7.3%	92.7%	72.0%	28.0%
	*p* < .001			*p* < .001		*p* < .001		*p* < .001	

However, only 5.5% of the Chinese LGB people surveyed said that medical care providers ever asked them about their sexual orientation when they were inquired about their sexual partners or sex life. Males (7.0%) were more likely than females (1.0%) to say that medical care providers ever asked them about their sexual orientation when they were inquired about their sexual partners or sex life (*p <* .001). People who were lesbian and gay (*p <* .001), between 25 and 39 (*p <* .001), and completed middle school or below (*p =* .02) were more likely to be asked about their sexual orientation by their medical care providers, whereas those who had an annual income of less than RMB10,000 (USD1,450) were less likely to be inquired about their sexual orientation (*p <* .001).

#### Negative treatment when receiving medical care services

About 8.0% of the Chinese LGB people surveyed reported having experienced negative treatment in medical care settings. The three most commonly reported negative treatments were (1) avoiding contact with them (2.0%), (2) being verbally attacked by medical staff, including ridicule, mockery, name-calling, derision, abuse, insult, etc. (0.9%), and (3) being unable to receive same quality medical treatment as other patients (0.7%).

Males (*p* = .002), those who identified as lesbian and gay (*p* < .001), those living in rural area (*p <* .001), those who were older (*p =* .001), and those who had lower education (*p <* .001), were more likely to report having experienced negative treatment in medical care settings and such differences were statistically significant.

#### Gender expression

Seventy percent (69.9%) of the Chinese LGB people surveyed said they restrained their gender expression to various degrees when receiving medical care services. Males (*p <* .001), lesbians and gay men (*p <* .001), those living in town and rural area (*p <* .001), those who were older (*p <* .001), and those who had lower level of education (*p <* .001) were more likely to say they restrained their gender expression to various degrees when receiving medical care services and such differences were statistically significant (see Table 2). In addition, those who had an annual income of less than RMB10,000 (USD1,450) were less likely to restrain their gender expression when receiving medical care services (*p <* .001).

### Mental health care

About 11.1% of LGB people had the experiences of receiving counselling/therapy (individually or in a group setting) regarding mental health. LGB people who were female (*p* = .04), pansexual (*p =* .01), between 25 and 39 (*p <* .001), lived in the city (*p <* .001), had college or above education (*p <* .001), and had higher level of income (*p <* .001), were more likely to report the experiences of receiving mental health care (see Table 3).

**Table 3:** Experiences of using mental health care services

	Mental health service utilization (*N* = 15,611)		Sexual orientation disclosure (*n* = 1738)*			Negative treatment (*n* = 1738)*		Suppress gender expression (*n* = 1738)*	
	Yes	No	Yes	It depends	No	Yes	No	Yes	No
Overall sample	11.1%	88.9%	42.8%	46.3%	11.0%	16.7%	83.3%	52.5%	47.5%
Gender									
Male	10.8%	89.2%	42.4%	45.1%	12.4%	16.0%	84.0%	56.2%	43.8%
Female	12.0%	88.0%	43.6%	49.2%	7.2%	18.6%	81.4%	43.0%	57.0%
	*p* = .04		*p* = .007			*p* = .20		*p* < .001	
Sexual orientation									
Lesbian / Gay	11.4%	88.6%	43.9%	44.7%	11.5%	18.2%	81.8%	53.5%	46.5%
Bisexual	9.7%	90.3%	37.9%	52.8%	9.3%	11.3%	88.7%	50.4%	49.6%
Pansexual	13.8%	86.2%	47.9%	47.9%	4.2%	9.9%	90.1%	38.0%	62.0%
Asexual	8.3%	91.7%	28.6%	57.1%	14.3%	17.9%	82.1%	50.0%	50.0%
Questioning	10.7%	89.3%	34.4%	50.0%	15.6%	12.5%	87.5%	62.5%	37.5%
	*p* = .01		*p* = .12			*p* = .04		*p* = .08	
Age group									
18–24	10.3%	89.7%	39.7%	49.0%	11.3%	15.1%	84.9%	53.0%	47.0%
25–39	12.9%	87.1%	48.9%	40.7%	10.3%	19.2%	80.8%	50.6%	49.4%
40 or above	11.4%	88.6%	31.6%	55.3%	13.2%	23.7%	76.3%	71.1%	28.9%
	*p* < .001		*p* = .003			*p* = .05		*p* = .04	
Living area									
City	11.8%	88.2%	42.5%	47.1%	10.4%	16.5%	83.5%	51.3%	48.7%
Town	8.4%	91.6%	45.5%	43.0%	11.5%	18.0%	82.0%	57.0%	43.0%
Rural area	8.9%	91.1%	41.0%	36.1%	23.0%	19.7%	80.3%	67.2%	32.8%
	*p* < .001		*p* = .03			*p* = .71		*p* = .02	
Education level									
Middle school or below	6.2%	93.8%	52.5%	35.0%	12.5%	27.5%	72.5%	47.5%	52.5%
High school	8.5%	91.5%	41.2%	43.2%	15.6%	20.1%	79.9%	56.3%	43.7%
College or above	11.9%	88.1%	42.7%	47.0%	10.3%	16.0%	84.0%	52.2%	47.8%
	*p* < .001		*p* = .13			*p* = .06		*p* = .45	
Income level									
Less than RMB10,000 (Less than USD1,450)	10.8%	89.2%	37.9%	48.9%	13.3%	15.9%	84.1%	54.9%	45.1%
RMB10,000 – RMB49,999 (USD1,450 – USD7,249)	10.5%	89.5%	45.6%	44.2%	10.2%	20.4%	79.6%	50.5%	49.5%
RMB50,000 – RMB99,999 (USD7,250 – USD 14,499)	10.9%	89.1%	47.2%	46.0%	6.8%	14.8%	85.2%	52.8%	47.2%
RMB100,000 or above (USD14,500 or above)	15.9%	84.1%	54.5%	38.2%	7.3%	15.2%	84.8%	44.9%	55.1%
	*p* < .001		*p* < .001			*p* = .15		*p* = .08	

Note. *the sample (*n* = 1738) includes participants who had the experiences of using mental health care

#### Sexual orientation disclosure

Among LGB people who have received mental health care, 42.8% said they would not be reluctant to disclose their sexual orientation to the mental health care providers, 46.3% said it depends, and only 11.0% said they would be reluctant. Those who were male (*p =* .007), 40 or above (*p* = .003), living in the more rural area (*p =* .03), and had higher level of income (*p <* .001) said they would be reluctant to disclose their sexual orientation to the mental health care providers.

#### Negative treatment when receiving mental health care services

Around 16.7% of those who have received mental health care said they had experienced negative treatment when receiving mental health services. The three most commonly reported negative treatments were (1) being advised by counsellors/doctors to change sexual orientation, gender identity or gender expression (7.8%), (2) being told that sexual orientation, gender identity or gender expression was the root of their mental health issues (5.9%), and (3) being recommended, coaxed into, or provided conversion therapy for sexual orientation, gender identity or gender expression (5.7%). Lesbians and gay men were more likely to report negative treatment when receiving mental health care (*p =* .04) compared with people of other sexual orientations.

#### Gender expression

About 52.5% of the respondents said they restrained their gender expression to various degrees when they received mental health care services. Those who were male (*p <* .001), older (*p =* .04), and living in the more rural area (*p* = .02), were more likely to say that they restrained their gender expression to various degrees when they received mental health care services.

## Discussion

The paper provides novel empirical evidence through the first larger-scale study to examine the health care utilization experiences among LGB people in China.

There are four findings that are particularly noteworthy and contributes to the understanding of LGB health.

First, contrary to the research literature that questions the willingness to disclose sexual orientation among Chinese LGB people in general because of cultural and familial pressure [25, 39], this study found that a high proportion of LGB people in this study were willing to disclose their sexual orientation to their health service providers. It was found that only 24.9% of the Chinese LGB people surveyed said they would not disclose to their doctor their sexual orientation if asked, 57.0% said it depends, whereas 18.1% said they would. Among LGB people who had received mental health care, 42.8% said they would not be reluctant to disclose their sexual orientation to the counsellors/medical staff, 46.3% said it depends, and only 11.0% said they would be reluctant. Thus, it debunks the myth that Chinese LGB people are necessarily unwilling to disclose their sexual orientation across all settings. Instead, many of them answered that ‘it depends’ – it is likely that ‘it depends’ on the attitudes that the health care providers display, including general attitudes and language used by the providers. Research has already identified several consistent recommendations pertinent to primary care settings for LGB-culturally competent care, including: guidance on inclusive clinical environments, standards for clinician-patient communication, sensitive documentation of sexual orientation, knowledge for cultural awareness, staff training, and addressing population health issues [40]. There is certainly scope for health care providers in China to proactively show that they are LGB-friendly to their clients, as the clients may assume their service providers to be LGB-unfriendly until proven otherwise.

Second, despite it was shown that Chinese LGB people were willing to disclose their sexual orientation to health care providers, this paper found that only 5.5% of the LGB people surveyed said medical care providers ever asked them about their sexual orientation when they were inquired about their sexual partners or sex life. Particularly, males were more likely than females to have been asked about their sexual orientation, revealing that sexual minority women were further invisibilized by the medical system. More research is needed to establish the reasons behind how sexual orientation is overlooked in health care settings. Such silencing may hamper the rapport building between the doctor and the patient. The non-disclosure of sexual orientation or presumed heterosexuality may even lead to insensitive questioning, incomprehensive evaluation, inappropriate disease screening, incorrect diagnosis, and inadequate treatment, which hinder the quality of the care [6]. As suggested earlier in this paper, medical and nursing professionals often lack adequate awareness, knowledge, and skills to work with LGB patients. There is room to systematically review the training that health care professionals in China receive about LGB issues, both in terms of on-the-job training as well as training in the academic and professional medical institutions.

Third, it was particularly worrying that 5.7% of the Chinese LGB people said in accessing mental health care services, they were recommended, coaxed into, or provided conversion therapy for sexual orientation. The American Psychological Association [41] clearly indicated that there is a lack of evidence regarding the efficacy of efforts to change sexual orientation. Such attempts may cause or exacerbate distress and poor mental health, including depression and suicidal thoughts. All forms of conversion therapy that are harmful to both physical and psychological health practiced against LGB people should be discontinued immediately. Mental health professionals should stop advising their clients for sexual orientation change. In the current era in which doctor-patient relationships in China are filled with tension, disputes, and conflicts [42–44], unprofessional actions taken by service providers can further alienate LGB people from the medical system in which they have little trust in the first place.

Fourth, the findings show that those who were younger, lived in the city, and had higher level of income were more likely to disclose their sexual orientation to their health care staff. In contrast, those who were male, 40 or above, living in the more rural area, and had higher level of income, said they would be reluctant to disclose their sexual orientation to the mental health care providers. It means that future cohorts of Chinese LGB people who are living in a country where rapid urbanization takes place and the GDP rises are likely to further demand the health services in their country to meet their needs and wants.

### Policy and practice implications

Given the prevalence of sexual orientation-related negative treatment, the findings suggest that LGB cultural competence can be further enhanced in the Chinese health care settings. Cultural competence is ‘a set of congruent behaviors, attitudes, and policies that come together in a system, agency, or among professionals and enable that system, agency, or those professionals to work effectively in cross-cultural situations’ [45]. In order to build a qualified workforce that addresses the health needs of LGB people [46], it is important to build cultural competence in medical and mental health care providers by enabling them to critically understand and analyse their personal and professional attitudes toward sexual and gender minorities [47]. In the latest health China strategy released on March 5, 2018, expanding universal health coverage and investment in the primary health-care workforce are the major clear policy goals [48]. Training and non-discrimination awareness raising shall be conducted for health care service providers at all levels in the medical systems in China. Also, specific modules on sexual orientation, gender identity and expression, and sex characteristics (SOGIESC) shall be included in medical curriculum. Service providers should be equipped with relevant knowledge and skills, to ensure clinical interactions are conducted in a respectful, affirming manner, for examples through using inclusive, gender-neutral language when communicating with patients and avoid assumptions about sexual orientation and gender identity [49]. Providing opportunities to disclose sexual orientation is integral to the provision of a quality and appropriate care [50]. Service providers should facilitate voluntary disclosure of sexual orientation (e.g., verbal communication and in-take forms), while at the same time respect that ‘coming out’ is an individual decision [50] especially in the Chinese context where ‘coming out’ is an especially complex phenomenon that involves negotiations in a familial context.

At hospitals, the management shall take the lead and ownership in promoting equality and diversity in the medical system. This can be achieved by creating a welcoming environment that is inclusive of LGB people (i.e., patients, visitors, and employees), improving health services and systems for LGB patient care and support, and through the cultivation of a diverse workforce by providing LGB-inclusive employee support and benefits [51]. Efforts should also be put to demonstrate positive commitment to the local LGB community. In China, there is an increasing number of non-governmental organizations that work with LGB people – they can be useful partners that medical service providers collaborate with.

It is also worth exploring whether LGB people may prefer LGB-specific services. A directory of LGBT-friendly medical services which ensure LGB people that staff members in such services have received a certain level of training about SOGIESC may be helpful.

The findings in this study show that Chinese LGB people were still being recommended, coaxed into, or provided conversion therapy for sexual orientation. At the national level, the Ministry of Health should not only formulate policies, regulations, clearly articulated codes of conduct, and transparent procedures and practices to ensure non-discrimination of LGB people in the health care system, but also pay particular focus on banning conversion therapy. In 2014, a landmark lawsuit case in China has ruled that ‘gay cure’ treatments as illegal. A gay man, Yang Teng, sued a ‘straight conversion’ clinic for attempting to cure him of homosexuality by treatments including hypnosis and electric shocks. The court rule in favour of Yang Teng and has clearly stated that homosexuality should not be perceived as a disease [28]. The National Health and Family Planning Commission should issue the necessary policies to prevent any institution or psychological professionals or therapists from forcing LGB people to undergo any kind of therapy that is offensive to the human dignity of LGB people.

### Limitations

While this study has significant empirical contributions and policy implications, a few limitations should be noted. First, the present study relied on non-probability sampling due to the absence of a sampling frame for LGB people in China. Although our sample was geographically scattered across China, the use of non-probability sampling might not yield a nationally representative sample, which posed limitations to the generalizability of the findings. The sample was very young and a majority of them were under the age of 40. In addition, our recruitment strategy might be biased toward those who had access to social media and were connected to the LGB community. Despite recruiting a large sample, the findings might be underrepresenting the voices of those who are disconnected with social media and the LGB community, who might face even more difficulty to gain access to relevant information and medical service. Moreover, this study focused only on LGB people’s self-reported experiences of health service and subjective biases may be involved.

### Future research

Further research shall explore the attitudes of Chinese health care service providers on LGB issues. In addition, it is important to explore the views of medical students on LGB issues, as they are the next generation of up-and-coming health care service providers. It is also important to understand the extent to which people who work in allied sectors, such as social work and education, are prepared to work with their LGB clients.

While this study has been quantitative in nature, future qualitative studies can be useful for eliciting the experiences of LGB people in health care settings in China. Future studies shall also explore the resilience and strengths that Chinese LGB people display in facing discrimination in medical services and the strategies that they use to deal with it. Research shall also study the experiences of Chinese LGB health workers who may themselves in turn be subject to discrimination because of their sexual orientation.

Further studies with nationally representative samples are needed for examining the experiences of physical and mental health care of LGB people, which can inform the development of health care policies and services in China. When nationally representative samples of LGB people are available in other parts of the world such as the US, it allows the health care experiences of heterosexual and LGB people be compared. Currently in the US, at least 12 federal government surveys collect data on sexual orientation, and 6 of those 12 surveys also collect data on gender identity. Of these 12 federal surveys, 10 are administered by the US Department of Health and Human Services and 2 are administered by the US Department of Justice. However, given the Chinese government’s increasingly tightening stance on LGB issues, it is unlikely that they would embark on any data collection of LGB people. Worse still, data collection on LGB people may be seen as a way to further monitor or even persecute LGB people in an authoritarian state.

Although this paper addresses understanding LGB people’s experiences of health care in China, it is also an example that shows the need to collect more evidence to understand LGB people’s experiences of health in diverse locales internationally. Such efforts would be most needed but also most challenging where social discrimination and persecution against non-heterosexual people is particularly pronounced in the former Soviet bloc countries of Eastern Europe and Central Asia, in sub-Saharan Africa, and in majority Arab and Muslim countries. World Health Organization [2] has highlighted that in countries where homosexuality is deemed a crime, health care professionals may be required by law to report the individuals concerned to the appropriate authorities, thereby effectively failing to meet the obligation of confidentiality incumbent on health care professionals. It is in these settings where LGB people’s health may be most at risk, and where further studies may be most needed.

## Conclusions

It has been estimated that there are at least 70 million gender and sexual minorities in China and their health care needs remain largely unaddressed by the current Chinese medical system. This study found that not only were LGB people invisibilized in the health care system, they were also subjected to various forms of negative treatment in medical and mental health care settings, and most notably conversion therapy. Given the prevalence of sexual orientation-related negative treatment, there is a strong need to enhance LGB cultural competence and sensitivity among health care providers. Policymakers in China should also formulate laws, policies, regulations, clearly articulated codes of conduct, and transparent procedures and practices to ensure equal rights and non-discrimination of LGB people in the health care system, with a particular focus on banning conversion therapy.

## Data Availability

The data and materials will not be made publicly available because of issues around confidentiality.

## Abbreviations

CCMD: Chinese Classification of Mental Disorders
LGB: Lesbian, gay and bisexual
LGBTI: Lesbian, gay, and bisexual, transgender and intersex
SOGIESC: Sexual orientation, gender identity and expression, and sex characteristics
UNDP: United Nations Development Programme
WHO: World Health Organization
